# Capturing intracellular oncogenic microRNAs with self-assembled DNA nanostructures for microRNA-based cancer therapy†
†Electronic supplementary information (ESI) available. See DOI: 10.1039/c8sc03039a


**DOI:** 10.1039/c8sc03039a

**Published:** 2018-08-08

**Authors:** Q. Liu, D. Wang, M. Yuan, B. F. He, J. Li, C. Mao, G. S. Wang, H. Qian

**Affiliations:** a Institute of Respiratory Diseases , Xinqiao Hospital , Third Military Medical University , Chongqing 400037 , China . Email: hqian@tmmu.edu.cn ; Email: wanggs@tmmu.edu.cn ; Fax: +86 23 65211653 ; Tel: +86 23 68755644; b Department of Chemistry , Purdue University , West Lafayette , IN 47907 , USA

## Abstract

Aberrantly overexpressed oncogenic microRNAs (miRNAs, miRs) are excellent targets for therapeutic interventions. Nevertheless, thus far, little progress has been made in developing miRNA-based drugs and techniques for clinical applications, especially for overexpressed miRNAs. In this study, we demonstrate that self-assembled DNA nanostructures bearing multiple DNA sequences that are complementary to a target miRNA can effectively capture the overexpressed oncogenic miRNA and subsequently inhibit cancer cell proliferation. Specifically, a DNA nanotube structure that carries functional DNA segments (single-stranded, duplex and hairpin forms) was designed and synthesized to capture two well-known overexpressed miRNAs, miR-21 and miR-155. It was found that all three DNA nanotubes significantly reduced both miRNA levels and inhibited cancer cell growth. Moreover, the capture efficiency was highly concentration dependent and was associated with the structural design of the DNA nanotube. These results demonstrate that through careful design, programmable DNA nanostructures can hijack the natural cellular machinery and can serve as nucleic acid drugs themselves. The concept of using self-assembled DNA nanostructures to disrupt the intracellular machinery for therapeutic purposes opens a new paradigm for exploiting self-assembled DNA nanostructures for miRNA-based anticancer therapy.

## Abstract 2

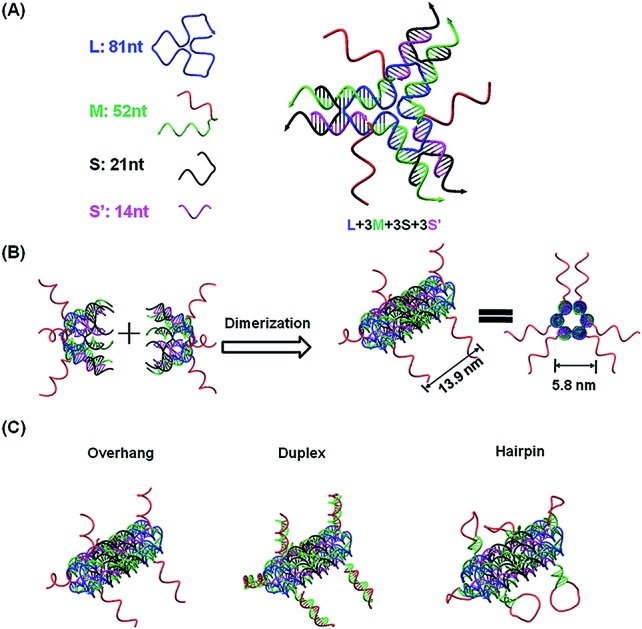
Aberrantly overexpressed oncogenic microRNAs (miRNAs, miRs) are excellent targets for therapeutic interventions.

## Introduction

Recently, miRNA-based therapeutics have been widely investigated and have shown great potential for cancer therapy. Based on their biological roles, cancer-related miRNAs are generally divided into two classes: tumor-suppressing miRNAs and oncogenic miRNAs. Thus, therapeutic interventions targeting miRNAs follow two strategies. One is to deliver synthetic miRNA mimics or analogs (tumor suppressor) to cells or tissues that express lower miRNA levels than their normal counterparts. The other strategy is to deliver viral or nonviral miRNA inhibitors to knockdown the aberrantly overexpressed oncogenic miRNAs. Much effort has been devoted to the delivery of miRNA mimics and analogs. Nonviral vectors, such as cationic polymers,^1^–^4^ dendrimers,^5^ liposomes,^6^–^8^ and inorganic particles,^9^,^10^ have been successfully explored as miRNA delivery vehicles. Indeed, restoring miRNA levels with these nanomaterials partially suppressed cancer cell proliferation and migration both *in vitro* and *in vivo*, implying that therapeutic interventions targeting miRNAs are promising and desirable. However, little attention is paid to developing novel nanomaterial platforms or strategies targeting aberrantly overexpressed oncogenic miRNAs. Currently, there are two main ways to knockdown miRNAs. One is to chemically modify short antisense oligonucleotides (antimiRs or ASON) to improve the stability and affinity of miRNA inhibitors, for instance, through 2-*O*′-methyl modification,^11^,^12^ peptide nucleic acid (PNA) antimiR^13^ or locked nucleic acid (LNA) technology;^14^,^15^ the other way is to load synthetic antimiR molecules into delivery vehicles to protect the vulnerable RNA cargo. For example, Kang *et al.* synthesized a biodegradable polymer nanocapsule to wrap antisense miR-21. The as-prepared nanocapsules were demonstrated to be an effective miRNA delivery platform and showed good tumor-suppressing capabilities *in vivo*.^16^ Moreover, Baptista *et al.* developed a DNA-based gold-nanobeacon system for the sensing and monitoring of intracellular miR-21.^17^,^18^ Slack *et al.* demonstrated that targeting the tumour microenvironment by silencing oncomiR miR-155 is a widely applicable nanotechnology for cancer therapy.^19^ Nevertheless, the above-mentioned methods need additional delivery vehicles to enable effective transportation of the nucleic acid drugs. Chemical modification of oligonucleotides is expensive, and the impact on the immune response and systemic toxicity remains unclear for practical applications. Therefore, it is highly desirable to engineer novel nanomaterials or develop new strategies to knockdown oncogenic miRNAs for miRNA-based cancer therapy.

Self-assembled DNA nanostructures have attracted increasing attention as novel functional materials in the field of nanomedicine due to their high programmability and biocompatibility and low cost. These structures have been explored as drug delivery vehicles for small molecules,^20^–^25^ imaging agents,^26^ and sensors^27^–^31^ and as aligning agents for a membrane protein study.^32^ Previously, we demonstrated that a DNA nanostructure exhibiting a “shuriken” shape can effectively deliver naked miR-145 mimics into colorectal cancer cells without the assistance of transfection reagents.^33^ Moreover, the DNA shuriken protected its cargo for a long enough time for it to exert tumor-suppressive effects in harsh physiological environments. Restoration of miR-145 greatly inhibited cancer cell growth *in vitro* and in a 3D cell culture system. Our study also indicated that the structural design is an important factor for DNA nanostructures as drug delivery vehicles. However, for intracellular overexpressed miRNAs, whether artificial DNA strands can compete with mature miRNA duplexes or miRNA-induced silencing complexes (miRISCs) and the underlying mechanism are rarely explored.

In this study, we propose that DNA nanostructures carrying multiple DNA segments complementary to overexpressed oncogenic miRNAs can capture target mature miRNAs effectively and subsequently inhibit cancer cell proliferation. As a proof-of-concept trial, DNA nanotubes with three different capturing units were designed and synthesized. The three capturing units were in single-stranded, duplex and hairpin forms. Two well-known overexpressed oncogenic miRNAs, miR-21 16,34,35 and miR-155,^36^ were selected as targets to validate the capture capability of the DNA nanotubes. Instead of acting solely as drug delivery vehicles or study tools, the functional DNA nanotubes designed in the current study can be considered as drugs themselves. The strategy we proposed here using designable functional DNA nanostructures as drugs to disrupt the natural cellular machinery for therapeutic purpose represents a step forward towards miRNA-based cancer therapy.

## Results and discussion

To capture mature miRNAs in the cytoplasm, the designed DNA nanostructure needs to meet at least two requirements. First, the capturing units, which are short DNA sequences, must be well-protected in the harsh intracellular environment for a long enough time for them to perform their functions. Second, the capturing units need to be accessible to miRNAs or ready to be released into the cytoplasm. The units can then compete with a mature miRNA duplex or miRISC.

**Fig. 1:**
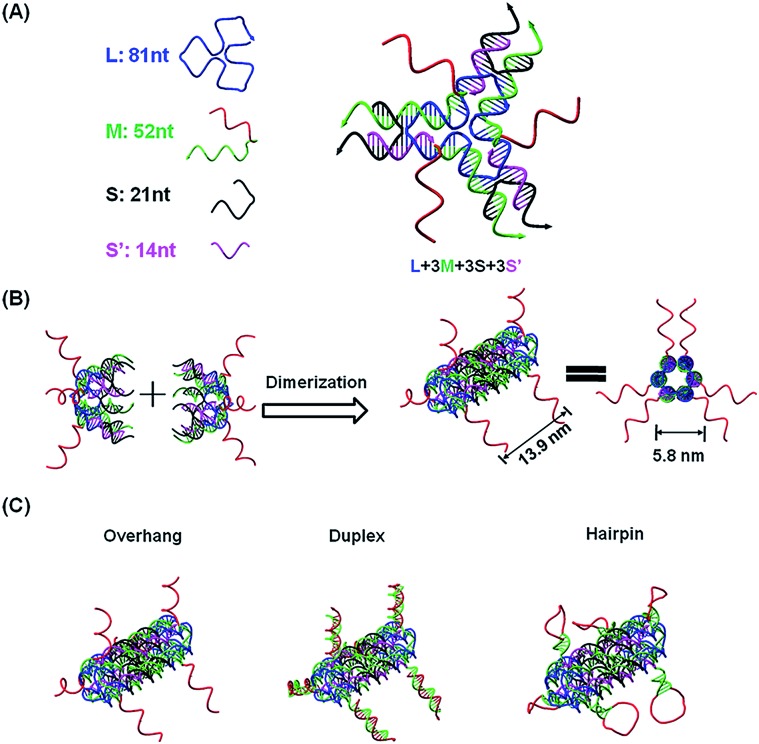
Functional DNA nanotube design. (A) Scheme of a DNA Y-motif design. The red DNA segment of strand M is the capturing unit in single-stranded form. (B) Two DNA Y-motifs bend by 90 degrees to form a nanotube structure. Each DNA nanotube carries 6 capturing units. The designed DNA nanotube is approximately 13.9 nm long and 5.8 nm wide (diameter). (C) DNA nanotubes carrying capturing units with different configurations: overhang, duplex and hairpin structures.

**Fig. 2:**
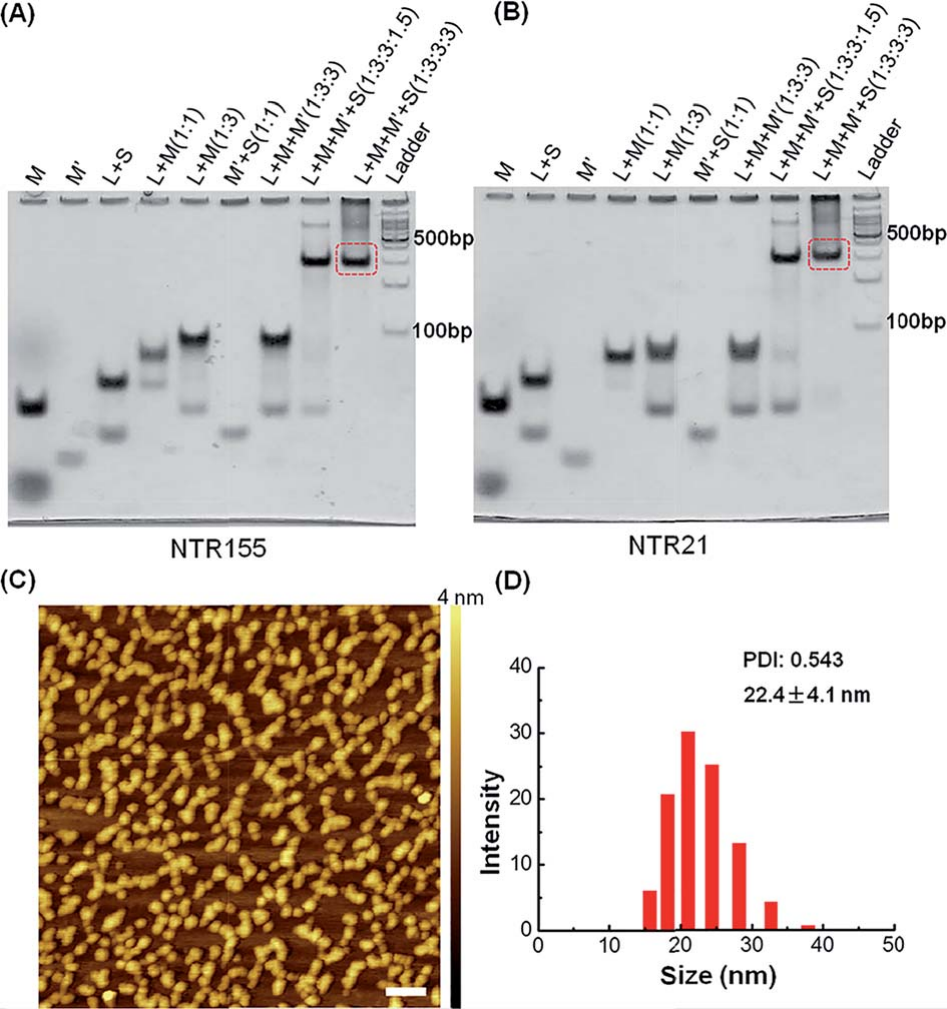
Characterization of DNA nanotubes with the overhang structure (NTR). (A) PAGE gel characterization of a DNA nanotube with a miR-155 capturing unit (NTR155). (B) Native PAGE analysis of a DNA nanotube with a miR-21 capturing unit (NTR21). (C) AFM imaging of NTR155. The image size is 500 nm × 500 nm (scale bar: 50 nm). (D) DLS measurement of NTR155. The DNA nanotube concentration for DLS measurements is 100 nM.

The current DNA nanotube design is modified from a previously reported DNA nanotube.^25^,^37^ Briefly, one core DNA L (blue), three identical M strands (green and red), S strands (black) and S′ strands (purple) form a Y-shaped motif (Fig. 1A, DNA Y-motif). The red region of strand M represents the functional units that target miRNAs. The three arms of the DNA Y-motif are identical, self-recognizable and highly flexible. Two DNA Y-motifs bend by 90 degrees and associate with each other to form a nanotube structure (Fig. 1B). The DNA nanotube is approximately 13.9 nm long and 5.8 nm wide. Each nanotube carries six functional units at its two ends (Fig. 1C). To study the structural effect of the functional DNA segments on the miRNA-capturing efficiency, the capturing units were designed in the overhang, hairpin or duplex configuration. The DNA oligos L, M, S and S′ were added together (1 : 3 : 3 : 3) and then subjected to a step-wise annealing procedure to achieve the nanotube formation. Fig. 2A and B show the native polyacrylamide gel electrophoresis (PAGE) analysis of DNA nanotubes with overhangs targeting miR-155 (NTR155) and miR-21 (NTR21), respectively. Both NTR155 and NTR21 formed a sharp, dominant band (indicated in the red box) on the gel with a yield of approximately 80%. The mobilities of NTR155 and NTR21 on the gel are consistent with their theoretical molecular weights (∼345 bp) and the 100 bp DNA ladder, indicating that this shape is indeed a nanotube structure instead of a Y-motif (∼172 bp) or other structures. The AFM imaging in Fig. 2C exhibited the morphology of the as-prepared DNA nanotubes. The nanotubes were evenly dispersed on a mica surface as discrete particles or small particle aggregates containing two or three individual nanotubes. This aggregation phenomenon is attributed to the drying process during sample preparation and the nonspecific interactions between the overhangs of different DNA nanotubes. The individual DNA nanotubes had a height of approximately 4 nm, which is consistent with their design. Dynamic light scattering (DLS) measurements further confirmed the formation and particle size of the DNA nanotubes, which were found to have a hydrodynamic diameter of 22.4 nm. Considering that the core structure of the DNA nanotube is 13.9 nm long and the six protruding overhangs of the DNA nanotube are ∼6.8 nm (21 bases) in length and highly flexible in three dimensions, it is reasonable to have an average hydrodynamic diameter of 22.4 ± 4.1 nm. DNA nanotubes with hairpin-shaped or duplex-form capturing units were also prepared and characterized by altering the overhang sequences (detailed sequence information can be found in the ESI†). Overall, we successfully synthesized and characterized a DNA nanotube with functionalities targeting miR-21 and miR-155. The obtained DNA nanotubes were demonstrated to have the right sizes and configurations.

**Fig. 3:**
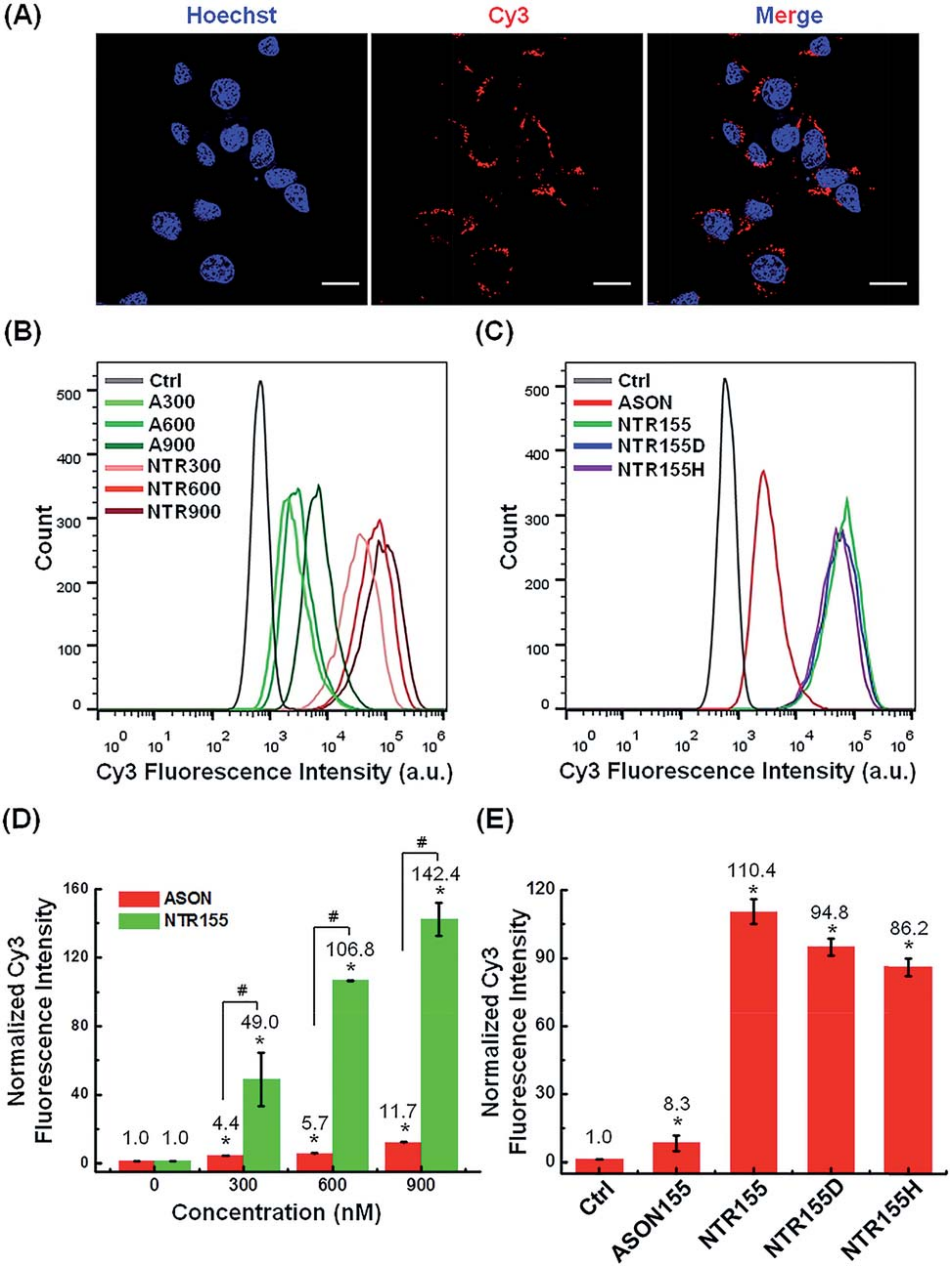
Cellular uptake study of functional DNA nanotubes targeting miR-155. (A) CLSM imaging of the internalized NTR155 in H1299 non-small cell lung cancer (NSCLC) cells. Cells were incubated with Cy3-tagged NTR155 (nanotube concentration: 50 nM; Cy3 fluorophore concentration: 300 nM) for 12 hours before imaging. The nucleus was stained with Hoechst 33342 (blue). Scale bar: 20 μm. (B) Flow cytometry analysis of the internalized NTR155 at different concentrations. A Cy3-tagged antisense oligonucleotide (ASON, miR-155 capturing unit) was also used as a control. The concentrations of ASON or capturing units of DNA nanotubes were 300 nM, 600 nM and 900 nM. The transfection time was 12 hours. (C) Flow cytometry evaluation of the transfection efficiency of all three types of capturing unit designs: single-stranded (NTR155), duplex (NTR155D) and hairpin (NTR155H). The capturing unit concentration used in this experiment was 300 nM. The transfection time was 12 hours. (D) Histograms showing the quantification of the internalized amount of DNA nanomaterials in (B). (E) Histograms showing the quantification of the internalized amount of DNA nanomaterials in (C). The data are representative of three separate experiments (mean ± S.E., *n* = 3) Student's *t*-test, *P*-values: *P < 0.05, ^#^*P* < 0.05.

Next, we evaluated the cellular internalization behaviors of DNA nanotubes using NTR155 for demonstration purposes. As shown in Fig. 3A, confocal laser scanning microscopy (CLSM) imaging revealed that Cy3-tagged NTR155 was substantially taken up by cells and aggregated as large particles (red) surrounding the nucleus. To more accurately evaluate the amount and internalization efficiency of the DNA nanotubes, fluorescence-activated cell sorting (FACS) was employed to quantify the cellular internalization under different experimental conditions. Fig. 3B shows the FACS plots of NTR155 with capturing unit concentrations from 300 nM to 900 nM. The same concentrations of antisense oligonucleotide DNA (ASON) complementary to miR-155 were also measured as controls. As expected, quantitative analysis indicated (Fig. 3D) that the red fluorescence intensities increased linearly with NTR155 concentration. However, the uptake efficiencies were much lower for ASON than NTR155 at every concentration point. The cellular uptake efficiencies of NTR155 were 11.1, 18.7 and 12.2 times higher than those of ASON at 300, 600 and 900 nM concentrations, respectively. We also investigated the effect of the capturing unit configuration of DNA nanotubes on their cellular uptake properties (Fig. 3C and E). All three types of DNA nanotubes, NTR155, and DNA nanotubes with duplex (NTR155D) and hairpin (NTR155H) configurations, had more pronounced and stronger internalized red fluorescence than ASON. However, there was no significant difference between the three DNA nanotubes. This result is probably due to the fact that the capturing units are relatively small compared with the core tubular structure. In addition, the size change of the three configurations is relatively small and may make a negligible contribution to the entire structure.

**Fig. 4:**
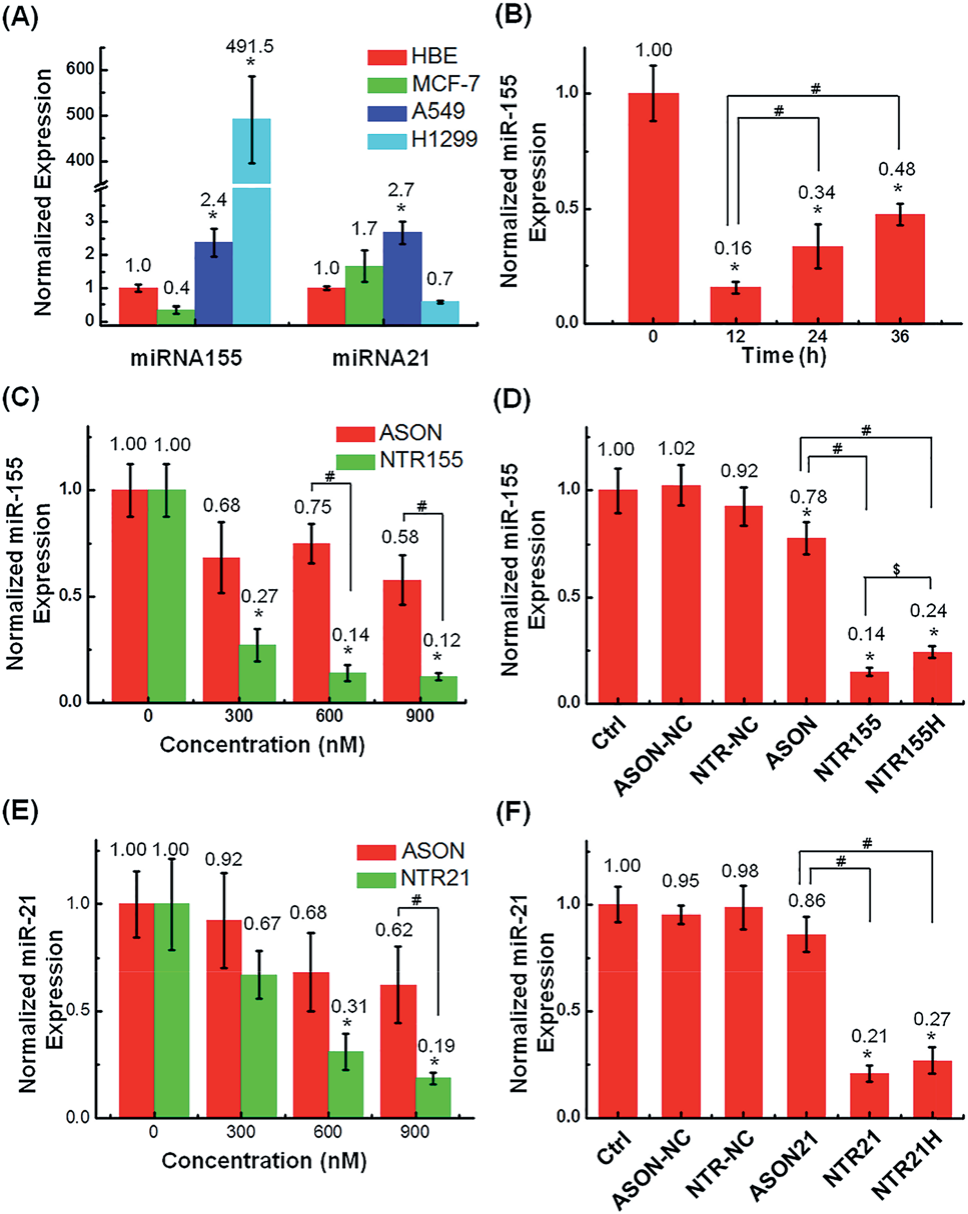
Capturing intracellular miRNAs with DNA nanotubes. (A) RT-qPCR screening of target miRNA expression levels in H1299 and A549 NSCLC cells, MCF-7 breast cancer cells and HBE human bronchial epithelial cells. U6 was set as the housekeeping gene. The expression levels of miR-155 and miR-21 in H1299 and MCF-7 cells were indeed upregulated. (B) Histograms showing the miR-155 levels in H1299 cells after they were treated with 50 nM NTR155 (300 nM capturing units) for 12, 24 and 36 hours after transfection. (C) Capturing efficiency of miR-155 at different nanotube and ASON concentrations. H1299 cells were treated with 50 nM, 100 nM and 150 nM NTR155 separately for 24 hours. Cells treated with ASON at concentrations of 300, 600 and 900 nM were also evaluated for comparison. The *X* axis represents the capturing unit concentration. (D) RT-qPCR evaluation of miRNA-155 levels after treatments with different DNA nanotubes. Cells were treated with 100 nM NTR155 and NTR155H and 600 nM ASON for 24 hours before RNA extraction. (E) Capturing efficiency of miR-21 in breast cancer cell line MCF-7 at different nanotube and ASON concentrations. The nanomaterial concentrations and incubation time were the same as described in (C). (F) RT-qPCR evaluation of miRNA-21 levels after treatments with different DNA nanotubes. The NTR21 and NTR21H concentrations were 100 nM. Data represent three separate experiments (mean ± S.E., *n* = 3) Student's *t*-test, *P*-values: *P < 0.05, ^#^ and ^$^ indicate significance at the *P* < 0.05 level for the connected groups.

The capturing capability of DNA nanotubes was evaluated using the RT-qPCR technique. We first screened miR-155 and miR-21 levels in H1299 and A549 NSCLC cells, MCF-7 breast cancer cells and HBE human bronchial epithelial cells. It was found that miR-155 was highly upregulated in H1299 cells and that miR-21 was overexpressed in MCF-7 cells (Fig. 4A). These results agree well with other reports about miR-155 and miR-21 38 expression profiles and indicate that the miR-155 and miR-21 in these two cell lines are indeed excellent targets to validate our concept for capturing intracellular miRNAs with DNA nanostructures. Fig. 4B shows the miR-155 levels in H1299 cells after treatment with 50 nM NTR155 for 12, 24 and 36 hours. Surprisingly, the miR-155 expression was reduced to a very low level of 16% at the 12 hour time point.

To assess the effect of NTR155 dose on its capturing efficiency, the NTR155 concentration was increased from 50 nM to 100 and 150 nM. ASON without the protection of a DNA nanostructure was also investigated for comparison. With capturing unit treatment concentrations of 300 nM, 600 nM and 900 nM, the miR-155 levels were reduced to 27%, 14% and 12%, respectively, by NTR155. This finding indicated that the capture of miR-155 by NTR155 is highly concentration dependent. In contrast, for ASON, the miR-155 levels were much higher than those of the NTR155-treated groups. We reasoned that a complex DNA structure that harbors a functional DNA sequence (capturing unit) is essential for successful miRNA knockdown. We further studied the effects of the capturing unit configurations on the capturing efficiency of miR-155. As shown in Fig. 4D, the overhang-structured NTR155 and the hairpin-structured NTR155H both significantly reduced the miR-155 level, compared with bare ASON. The capturing efficiency was higher for NTR155 than NTR155H in the current experimental setting. This finding suggested that the capturing unit configuration indeed affects intracellular miRNA binding. Unfortunately, the capturing efficiency of NTR155D (capturing unit in the duplex form) was severely disrupted in the qPCR process and was not suitable for comparison in this experiment. To assess the applicability of capturing intracellular miRNAs with self-assembled DNA nanostructures, we further validated the concept with another well-known oncogenic miRNA target, miR-21, in MCF-7 cells. Fig. 4E shows the miR-21 levels of MCF-7 cells after incubation with NTR21 and ASON at different concentrations. Similarly, the capturing efficiency of NTR21 was highly concentration dependent, and bare ASON targeting miR-21 had a worse knockdown effect than NTR21.

The structural effect of the capturing unit for miR-21 knockdown seemed to be less significant than for miR-155 in H1299 cells (Fig. 4F). Although the miR-21 level of the NTR21-treated group was always lower than that of its NTR21H-treated counterpart in quadruplicate experiments, the difference between these two groups was not always significant at the *P* = 0.05 level.

**Fig. 5:**
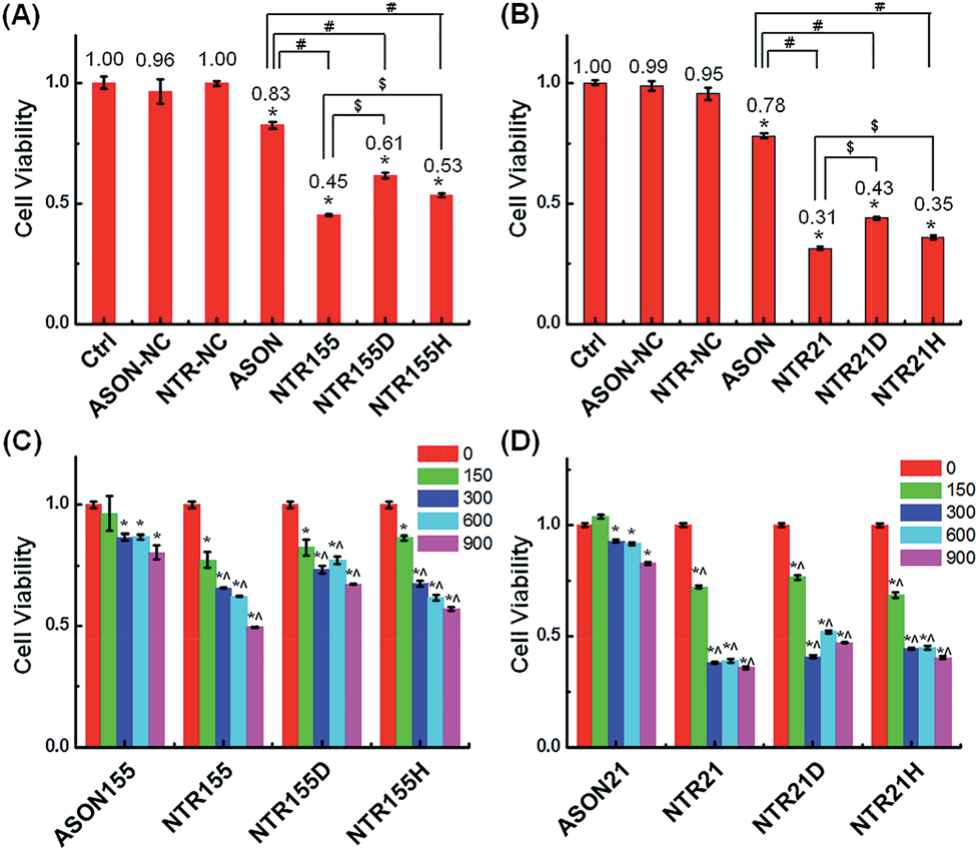
Study of the anticancer effects of capturing intracellular miRNAs. (A) Cell viability of cells treated with ASON, NTR155, NTR155D and NTR155H in NSCLC cell line H1299. The capturing unit concentration was kept at 600 nM (100 nM for DNA nanotubes), and the incubation time was 24 hours. (B) Cell viability of H1299 cells treated with ASON, NTR21, NTR21D and NTR21H. MCF-7 cells were subjected to the same experimental procedure as described in (A). (C and D) The suppressed cell proliferation effects of different DNA nanotubes and doses to target miR-155 and miR-21. The incubation time was 24 hours. Data represent five separate experiments (mean ± S.E., *n* = 5) Student's *t*-test, *P*-values: *P < 0.05, ^#^ and ^$^ indicate significance at the *P* < 0.05 level for the connected groups.

Aberrantly overexpressed intracellular miRNAs are strongly associated with a variety of diseases, especially cancer. For example, miR-21 is a famous oncogenic miRNA in breast cancer and is intensively investigated as a therapeutic target.^39^ miR-155 was reported to be overexpressed in NSCLC and associated with cell proliferation by targeting FoxO1 and other genes.^40^–^43^ Thus, capturing intracellular overexpressed miR-21 and miR-155 might upregulate their target genes and subsequently alter the cell fate and cancer growth. To explore the potential of capturing miRNAs with functional DNA nanostructures for miRNA-based therapeutics, we evaluated the anticancer effect of functional DNA nanotubes by MTT. Fig. 5A revealed that the cell viabilities of ASON-, NTR155-, NTR155D- and NTR155H-treated H1299 cells were 83%, 45%, 61% and 53%, respectively. Notably, NTR155 had a better killing effect on cancer cells than NTR155H, which is consistent with the miRNA knockdown results in Fig. 4D. Thus, it is rational to infer that the miRNA capturing efficiency of NTR155D is lower than that of NTR155 or NTR155H. Similarly, in the case of miR-21, NTR21 had the best anticancer capability among all sample groups (Fig. 5B). The trend of cell viabilities after treatment with NTR21, NTR21D and NTR21H implied that NTR21 also has the best configuration of the capturing unit for capturing intracellular miR-21 and suppressing cancer cell proliferation. It is worth noting that the cancer cell growth inhibitory effects of ASON were much worse than those of self-assembled functional DNA nanotubes for both miR-21 and miR-155. This finding again demonstrated that although the capturing units are vulnerable DNA molecules in nature, the presence of a core DNA nanotube structure protects the capturing units against enzymatic digestion in physiological environments (Fig. S2†). This phenomenon laid a solid foundation for the future use of self-assembled DNA nanostructures themselves as multifunctional, cost-effective anticancer drugs.

**Fig. 6:**
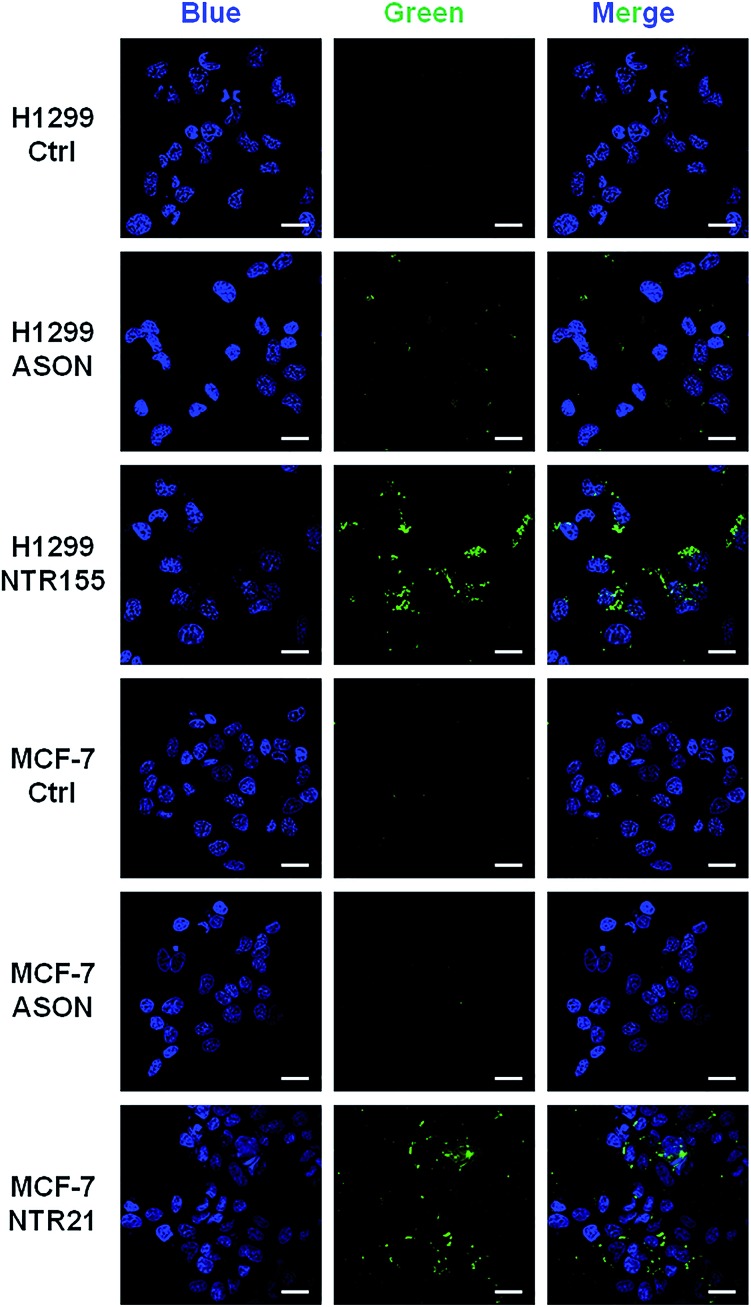
Morphological analysis of the cell viability with targeting of miR-21 and miR-155. Cells were treated with 100 nM NTR21 and NTR155 and 600 nM ASON for 24 hours. The cells were then stained with Hoechst (blue color) and Cell Dead Green (green color) before imaging. Scale bar: 20 μm.

To more directly observe the anticancer effects rendered by capturing intracellular overexpressed miRNAs, a Dead Cell Green assay and microscope imaging were employed for a morphological evaluation of cancer cells. As shown in Fig. 6, cells were incubated with NTR (or ASON) for 24 hours and then stained with Dead Cell Green. Dead cells with permeable membranes were stained green. As expected, NTR155-treated H1299 cells and NTR21-treated MCF-7 cells exhibited strong green fluorescence, indicating the existence of a large population of dead cells. In contrast, for ASON-treated cells, there were also a small fraction of dead cells, which is consistent with its miRNA knockdown efficiency in Fig. 4 and 5.

Recently, miRNA-based anticancer therapeutics have received increasing attention. Compared with the delivery of synthetic tumor-suppressive miRNA molecules or analogs, nanotechnology and drugs targeting overexpressed oncogenic miRNAs are less investigated. The use of viral vectors or plasmids to sponge miRNA is limited in laboratory studies. There were a few studies reported that used polymers to wrap and deliver antagomir molecules to knockdown intracellular overexpressed miRNAs.^16^ The delivered antisense miRNA molecules indeed silenced the target miRNAs *in vitro* and *in vivo*. Nevertheless, the functional group used in these studies was an RNA molecule, which is expensive and highly active and may cause side effects once taken up by cells. In this study, we approached this issue from a different perspective. Overexpressed, mature miRNAs exist in the cytoplasm either in duplex form or as part of the miRISC. Mother nature has developed sophisticated machinery for miRNAs to fulfil their tasks in cells. We used self-assembled DNA nanotube structures bearing differently shaped capturing units to disrupt this miRNA functional network by hijacking natural miRNAs from the native duplex form or RISC. The capturing units were simply DNA sequences in different configurations, making them cheap and effective. Although the detailed capturing dynamics need to be further investigated, the DNA nanotubes with miRNA capturing units in this study indeed silenced the miR-155 and miR-21 targets and subsequently inhibited cancer cell proliferation. Additionally, the configuration of the capturing units is an essential factor in the capturing efficiency. In the current study, the DNA nanotubes with capturing units in the overhang form have a better capturing efficiency than hairpin-shaped or duplex form DNA nanotubes. This is probably due to the fact that the overhang structure is the easiest configuration to gain access to target miRNAs. On the other hand, the overhang structure is less protected in the cytoplasm compared with the hairpin or duplex structure. As shown in Fig. 4B, the miR-155 level increased from the 12 h to the 48 h time point, indicating that the capturing units in NTR155 were either degraded or insufficient to suppress target miRNAs. Thus, the configuration of capturing unit is an important factor in designing DNA nanostructures for capturing miRNAs. For example, the use of a hairpin or duplex structure may provide a slower rate to open its structure and then capture the target miRNAs. This dynamic difference may render sustained capturing unit release and maintain a lower target miRNA level for practical applications.

## Conclusions

In conclusion, we designed and synthesized self-assembled DNA nanotubes bearing DNA sequences that target intracellular overexpressed miR-155 in NSCLC cells and miR-21 in MCF-7 breast cancer cells. The functional units of DNA nanotubes were designed to have the single-stranded overhang, duplex and hairpin forms. It was found that all three types of DNA nanotubes can effectively knockdown intracellular miRNA levels and subsequently inhibit cancer cell proliferation. The capturing efficiency of DNA nanotubes with overhang capturing units is higher than that of DNA nanotubes with hairpin and duplex units. Our results in this study suggest that DNA nanostructures with rationally designed functionalities can serve as nucleic acid drugs themselves. The concept we proposed here of hijacking the cellular miRNA machinery through carefully designed DNA nanostructures is highly promising for cancer therapy targeting overexpressed oncogenic miRNAs.

## Conflicts of interest

There are no conflicts of interest to declare.

## Supplementary Material

Supplementary information
