# Supplementary material for: Capturing intracellular oncogenic microRNAs with self-assembled DNA nanostructures for microRNA-based cancer therapy
Source: Chem Sci. 2018 Aug 8;9(38):7562–8. doi: 10.1039/c8sc03039a (PMC6180306; doi:10.1039/c8sc03039a)
Supplement: Supplementary file 1 [file SC-009-C8SC03039A-s001.pdf]

Electronic Supplementary Material (ESI) for Chemical Science.
This journal is © The Royal Society of Chemistry 2018

Supporting Information

Capturing intracellular oncogenic microRNAs with self-assembled DNA nanostructures for microRNA-based cancer therapy

Q. Liu,[a] D. Wang,[a] M. Yuan,[a] B. F. He,[a] J. Li,[a] C. Mao,[b] G. S. Wang[a,*] and H. Qian[a,*]

Experimental methods

1.1 Materials and Reagents

The DNA sequences of the DNA nanotube were designed using the computer program SEQUIN. All DNA strands (PAGE purified) were purchased from Sangon Biological Engineering (Shanghai, China) and used as received. Strand L: 5'-GTA GGt TTT TTC TTG CCA GGC ACC ATC GTA GGT TTT TTC TTG CCA GGC ACC ATC GTA GGT TTT TTC TTG CCA GGC ACC ATC-3'; M(155): 5'-ACC CCT ATC ACG ATT AGC ATT AAT TGC AAG CCT ACG ATG GAC ACG GTA ACG AC-3'; M(155D): 5'-ATG CTA ATC GTG ATA GGG GT TT GCA AGC CTA CGA TGG ACA CGG TAA CGA C-3'; M(155H): 5'-GCA GCT ACC CCT ATC ACG ATT AGC ATT AAA GCT GCG TGC AAG CCT ACG ATG GAC ACG GTA ACG AC-3'; M(21)-NC: 5'-TTT GTA CTA CAC AAA AGT ACT GAA GCA AGC CTA CGA TGG ACA CGG TAA CGA C-3' ; M(21): 5'-TCA ACA TCA GTC TGA TAA GCT ATT GCA AGC CTA CGA TGG ACA CGG TAA CGA C-3'; M(21D): 5'-CTT ATC AGA CTG ATG TTG ATT GCA AGC CTA CGA TGG ACA CGG TAA CGA C-3'; M(21H): 5'-AGC AGC TCA ACA TCA GTC TGA TAA GCT AGC TGC T-3'; M(21)-NC: 5'-CAG TAC TTT TGT AGT ACA A**AA** GCA AGC CTA CGA TGG ACA CGG TAA CGA C-3' ; M': 5'-TAG CAA CCT GCC TG-3'; S: 5'-ACC GTG TGG TTG CTA GTC GTT-3'; fluorophore-modified S: 5'-Cy3-ACC GTG TGG TTG CTA GTC GTT-3'; antagomiR155 (ASON155): 5'-ACC CCT ATC ACG ATT AGC ATT AA-3'; antagomiR155-NC: 5'-TTT GTA CTA CAC AAA AGT ACT G-3'; fluorophore-modified antagomiR155 (F-ASON155): 5'-Cy3-ACC CCT ATC ACG ATT AGC ATT AA-3'; antagomiR21 (ASON21): 5'-TCA ACA TCA GTC TGA TAA GCT ATT-3'; antagomiR21-NC: 5'-CAG TAC TTT TGT AGT ACA A-3' and fluorophore-modified antagomiR21 (F-ASON21): 5'-Cy3-TCA ACA TCA GTC TGA TAA GCT ATT-3'. Acryl/bis 40% Solution (19:1), Tris base, EDTA-Na, acetic acid, magnesium acetate, formamide, 100 bp DNA Ladder and Hoechst 33342 were acquired from Sangon Biological Engineering (Shanghai, China). Stains-all and propidium iodide (PI) were obtained from Sigma Aldrich (USA). LysoTracker green DND-26 was purchased from Life Technologies Corporation (USA). Modified Eagle's Medium (MEM), RPMI-1640 medium, fetal bovine serum (FBS) and trypsin (0.25%) were obtained from Gibco (USA). MTT Cell Proliferation and Cytotoxicity Assay Kit was acquired from Beyotime Biotechnology (Shanghai, China). A reverse transcription polymerase chain reaction (RT-PCR) kit and LIVE/DEAD™ Fixable Dead Cell Stain Kit were purchased from Thermo Fisher Scientific (USA). X-tremeGENE HP DNA Transfection Reagent and FastStart Essential DNA Green MasterMix were purchased from Roche (Basel, Switzerland). Total miRNAs were collected using a Qiagen RNeasy mini kit (Germany).

1.2 Synthesis and characterization of DNA nanotubes

The component DNA strands L, M, M' and S were mixed together at a molar ratio of 1:3:3:3 in Tris-Acetic-EDTA-Mg^{2+} (TAE/Mg^{2+}) buffer (pH 8.0) and then subjected to the following annealing process: 95°C/5 min, 65°C/30 min, 50°C/30 min, 37°C/30 min, and 22°C /30 min. After the annealing, the DNA strands formed a nanotube carrying 6 overhangs/hairpin that should hybridize to the miR-155/miR-21 strands in cells. The nanotube with the duplex functional group was first synthesized as mentioned above, but the obtained nanotube was further mixed with AntigomiR 155/21 at a molar ratio of L:M:M':S:AntagomiR = 1:3:3:3:3 to form the final conformations at room temperature for 30 min.

The DNA nanotubes were analyzed by 6% native polyacrylamide (diluted from 40% 19:1 acrylamide/bis acrylamide) gel electrophoresis (PAGE), which was run on a Bio-Rad Electrophoresis System (USA) at room temperature for 1 hour at a constant voltage of 120 V. For running buffer, 1× TAE/Mg^{2+} was used, and the gel was stained with Stains-all solution (0.01%). The DNA nanotube solution was diluted to ~100 nM and measured by DLS (Malvern Instruments Laser Target Designator, UK) in 1× TAE/Mg^{2}+ buffer. Pure 1× TAE/Mg^{2+} was used as a blank.

For AFM imaging, 5 μL DNA sample solution was dropped onto a freshly cleaved mica surface and incubated for 2 min to allow for strong adsorption. The sample drop was then washed off using 50 μL 2 mM $Mg(Ac)_2$ solutions and dried by compressed nitrogen. The DNA nanotubes were then imaged by AC-mode AFM using an Agilent 5500 AFM system (Agilent Technologies, AZ, USA) with NSC15 tips (silicon cantilever, MikroMasch, tip radius: 8 nm). The tip-surface interaction was minimized by optimizing the scan set-point.

1.3 Cell culture and cellular uptake of DNA nanotubes

H1299 cells were cultured in RPMI-1640 medium; MCF-7 cells were cultured in Modified Eagle's Medium (MEM). The media all contained 10% fetal bovine serum (FBS), 100 units mL^{-1} of penicillin and 100 μg mL^{-1} of streptomycin. These cells were grown in a 95% humidified atmosphere of 5% CO_2 at 37°C. The cells were trypsinized and counted before each experiment when they grew to 80%-90% confluence.

The internalization of DNA nanotubes was observed with a confocal laser scanning microscope (Leica, Germany) and flow cytometry (Backman, Gallios, USA). For confocal microscopy, cells were cultured in a 24-well plate with a coverslip over each well overnight at a density of 1×10^4 cells/cm^2 under standard culture conditions. Then, the cells were treated with Cy3-labeled DNA nanotube (strand S was replaced with Cy3-labeled strand S) and transfection reagent for 12 hours. The minimum volume ratio between the DNA sample and medium was 1:8. After the treatment, the cells were stained with Hoechst 33342 dye (final concentration: 1 μg mL^{-1}) for 30 min, washed with PBS buffer 5 times and fixed with a mixture of 4% paraformaldehyde for 15 min. Finally, the cells were washed with PBS buffer 3 times before imaging. For live imaging, cells were cultured in 35 mm glass bottom dishes (Nunc) overnight at a density of 2×10^4 cells/cm^2 and treated with DNA nanotubes and Hoechst 33342 as above. Fluorescence images were obtained with a 40× oil-immersion objective lens, excitation wavelengths of 405 nm and 543 nm, and emission wavelengths

between 430/480 nm and 550/630 nm for Cy3 and Hoechst 33342, respectively.

For flow cytometry assays, cells were cultured in a 12-well plate overnight at a density of 2×10^4 cell/cm^2 and transfected with Cy3-labeled DNA nanotubes for 12 hours. After being washed 3 times with PBS buffer, the cells were digested by trypsin and collected in PBS buffer with 2% FBS. The collected cells were centrifuged at 1000 rpm for 10 min and washed with PBS buffer 2 times. Finally, the cells were resuspended in 350 µL of PBS buffer and used for flow cytometry.

1.4 Cell viability determination by MTT assay

Cells were cultured in a 96-well plate overnight with a seeding density of 1×10^3 cells/well. Then, the cells were treated with three kinds of DNA nanotubes at different concentrations with transfection reagent for 12 hours. Thereafter, the cells were cultured in fresh medium for 48 hours. Finally, the viability was determined by the MTT assay.

1.5 RT-qPCR analysis of miRNA expression

RT-qPCR was utilized to quantify the miRNA level. Cells were cultured and treated exactly as previously described except that they were cultured in a 6-well plate. After transfection with DNA nanotubes, the cells were incubated in fresh medium for 24 hours and then washed 3 times with PBS. Total miRNAs were collected using a RNeasy mini kit (Qiagen). cDNAs were obtained using a reverse transcription polymerase chain reaction (RT-PCR) kit (Thermo Fisher Scientific) following the manufacturer's instructions. Real time-qPCR (RT-qPCR) was carried out on a Bio-Rad CFX 96 real-time PCR system (USA) by using FastStart Essential DNA Green MasterMix. Each sample was duplicated, and U6 was used as an internal control. The primers for reverse transcription and qPCR measurement are described below: miRNA 155 reverse transcription primer: 5'-CTC AAC TGG TGT CGT GGA GTC GGC AAT TCA GTT GAG ACC CCT AT-3'; miRNA 155 forward primer: 5'-ACA CTC CAG CTG GGT TAA TGC TAA TCG TG-3'; miRNA 21 reverse transcription primer: 5'-CTC AAC TGG TGT CGT GGA GTC GGC AAT TCA GTT GAG TCA ACA TC-3'; miRNA 21 forward primer: 5'-ACA CTC CAG CTG GGT AGC TTA TCA GAC T-3'; miRNA reverse primer (for both miRNA 155 and miRNA 21): 5'-CTC AAC TGG TGT CGT GGA GT-3'; U6 reverse transcription primer: 5'-GTC GTA TCC AGT GCA GGG TCC GAG GTA TTC GCA CTG GAT ACG ACA AAA TA-3'; U6 forward primer: 5'-AGA GAA GAT TAG CAT GGC CCC TG-3'; and U6 reverse primer: 5'-AGT GCA GGG TCC GAG GTA TT-3'.

1.6 Morphology analysis of cell viability

Bright field images of the cell morphology were captured by an inverted microscope (Nikon ECLIPSE TS100) with a 20× air objective lens. LIVE/DEAD™ Fixable Dead Cell Stain Kit (green) was applied to study the cell viability according to the manufacturer's instruction. Green fluorescence images were obtained by using a confocal laser scanning microscope (Leica, Germany) with an excitation wavelength of 488 nm and emission wavelength between 495 nm and 535 nm. The cell death level was determined by flow cytometry (Backman, Gallios, USA) after cells were stained with propidium iodide (PI). Cells were treated and collected as mentioned in Section 1.3 for flow cytometry study except that the DNA nanotubes were not fluorescently labeled. Afterward, cells were treated with PI for 15 min, washed with PBS buffer 2 times and detected by flow cytometry immediately.

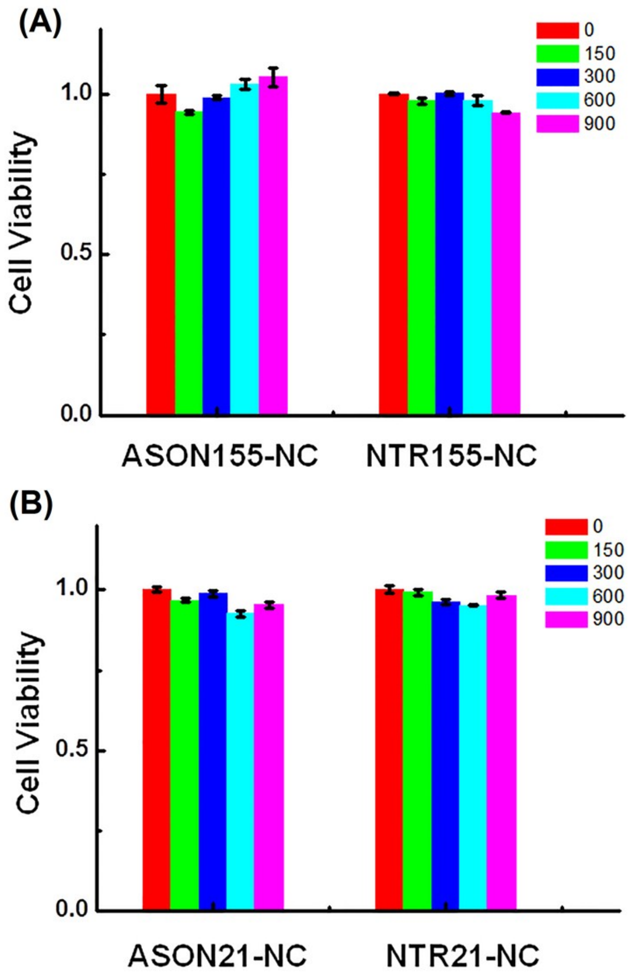

Fig. S1 The cell viability of scrambled ASON and DNA nanotube treated cells. All NC groups showed no toxicity at all doses from 0 to 900 nM. (A) Scrambled ASON and DNA nanotube targeting miR-155 in NSCLC cell line H1299 and (B) targeting miR-21 in Breast cancer cell line MCF-7. Data represent five separate experiments (mean ± S.E., n = 5) Student's *t*-test, *P*-values: $^*P < 0.05$,

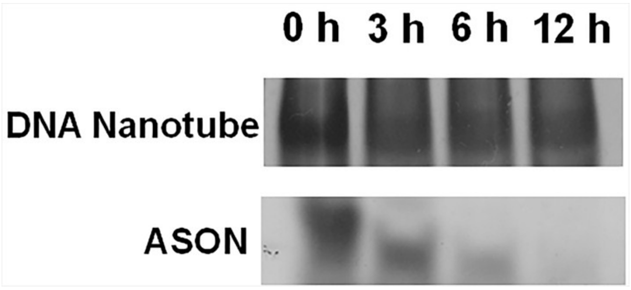

Fig. S2 Native PAGE gel showing the DNA nanotube and ASON incubated with 10% FBS at 37 °C for different times.

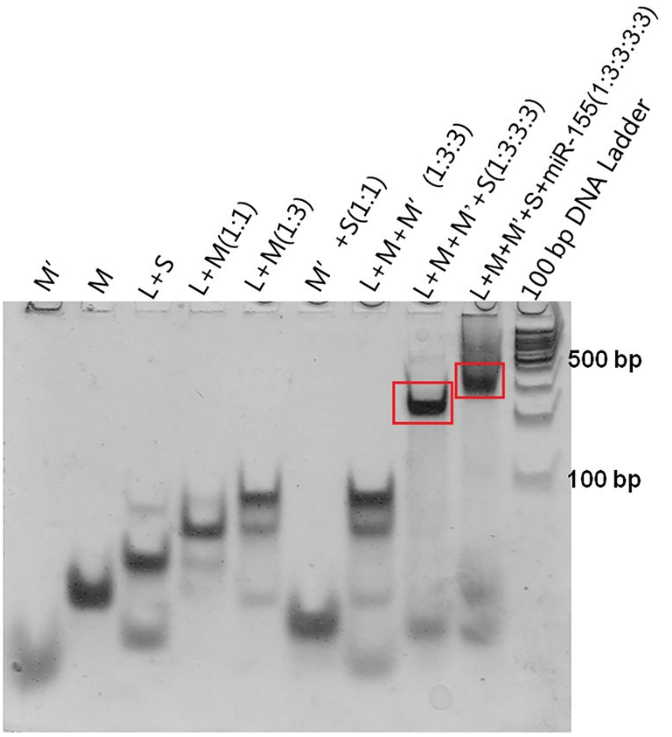

Fig. S3 Native PAGE gel showed the formation of DNA nanotube carrying miRNA cargo. Different combinations of individual DNA strands were mixed and annealed regularly. DNA nanotube carrying six miR-155 strands showed a clear shift compared with bare DNA nanotube in the gel.
